# Ergonomics in Laparoscopic Box Training and Virtual Reality (VR) Simulations: A Systematic Review

**DOI:** 10.7759/cureus.76107

**Published:** 2024-12-20

**Authors:** Md Rezaul Karim, Sola Kim, Rafiza Islam, Amos Kong, Bijendra Patel

**Affiliations:** 1 Department of Surgery, The Royal London Hospital, London, GBR; 2 Department of Surgical Science, Barts Cancer Institute, Queen Mary University of London, London, GBR; 3 Department of General Surgery, Ealing Hospital, London, GBR; 4 Department of Surgical Science, University of Leicester, London, GBR

**Keywords:** ergonomics, laparoscopic box training, laparoscopic surgery, laparoscopic training, laparoscopic vr training, surgical-education, surgical simulation

## Abstract

Laparoscopic surgery has now become the gold standard in managing most surgical cases. Despite its advantages, working hours and in-theatre training restrictions have prompted trainees to explore alternatives like virtual reality (VR) simulations and box training. Furthermore, given the increased frequency of minimally invasive surgery and the prevalence of musculoskeletal issues among surgeons, there’s a growing effort to optimize ergonomics. There is currently a lack of focus on ergonomics in training, prompting our study to inspect VR and box training's ergonomic outcomes and their impact on task performance.

This systematic review was conducted at Barts Cancer Institute, Queen Mary University, from January 27, 2024, to April 22, 2024. Multiple databases were searched electronically from January 1980 to March 2024, including only articles that utilized electromyography (EMG) or the NASA Task Load Index (NASA-TLX) to measure ergonomics. Two reviewers independently conducted eligibility assessment, data extraction, and risk of bias evaluation, with discrepancies resolved through consensus discussions.

Ultimately, 12 studies were selected. EMG results indicated a higher %MVC (maximum voluntary contraction) in box training in poor ergonomic settings and a negative correlation between skills and muscle activity. Both modalities showed a significant decrease in NASA-TLX scores after training on the simulators and when comparing novice and experienced surgeons. Furthermore, a reduction in physical demand and improved task performance was observed, with significant differences found between experts and novices in a VR appendicectomy scenario.

This systematic review reveals reduced muscle activity and physical demand among trained individuals in laparoscopic surgery with both box trainers and VR simulators, emphasizing the importance of addressing ergonomic considerations. To advance our understanding of surgical ergonomics, the standardization of measurement methods and higher-quality evidence, particularly through randomized controlled trials, are recommended.

## Introduction and background

Minimally invasive surgery has emerged as the cornerstone of therapeutic interventions for a wide range of medical conditions. Following the acceptance of laparoscopic surgery, restrictions in working hours have prompted many trainees to explore alternative modalities to hone their laparoscopic skills.

Alternatives include virtual reality (VR) simulations, box training exercises, and other simulation-based and wet lab-based laparoscopic training. Box trainers offer a cost-effective and portable solution, enabling training opportunities around the clock. Conversely, VR simulators, a pricier alternative, constitute a computer-based training apparatus. Various studies have shown improvements in technical skills, reduced time taken to perform procedures, and fewer errors after training sessions in both modalities [1,2].

While laparoscopic surgery has gained favor within the surgical community due to its benefits - such as decreased pain, reduced complication rates, and shorter hospital stays [3] - it remains imperative to acknowledge a significant drawback: the lack of ergonomic optimizations.

Suboptimal positioning during training and procedures can lead to reduced work capacity and performance. Additionally, Cuschieri has described “mental exhaustion, impaired surgical judgment, and reduced dexterity” resulting from ergonomic strains in laparoscopy [4]. Consequently, studies have sought to determine the optimal positions for trainees during training sessions. Recommendations include maintaining a monitor distance of 4-8 ft and positioning the screen below eye level for a “gaze-down” view [5,6].

Studies have also documented enhanced task performance and training efficiency in settings where ergonomic simulations are implemented, even in a skills lab environment. It’s important to note that, within these trials, sample sizes are often limited, and the selected tasks may not reflect complex surgical procedures, warranting caution in generalizing results.

To our knowledge, no systematic review has specifically examined ergonomic outcomes in laparoscopic training. Therefore, our primary aim is to assess the ergonomic outcomes and musculoskeletal strain in individuals undergoing laparoscopic surgery training with box trainers or VR simulations. Additionally, we aim to explore the impact of both training modalities on task performance by reviewing electromyography (EMG) and the NASA Task Load Index (NASA-TLX) results.

## Review

Materials and methods

Study Design

This systematic review was conducted by two independent reviewers at Barts Cancer Institute, Queen Mary University of London, from January 27, 2024, and was completed by April 22, 2024.

The protocol of the systematic review was prospectively registered on the Prospective Register of Systematic Reviews (PROSPERO). The registration number is CRD42024529079 (https://www.crd.york.ac.uk/prospero/display_record.php?RecordID=529079).

Our systematic review consisted of individuals undergoing laparoscopic training with either box trainers or VR simulators. The primary outcomes being studied consisted of NASA-TLX and EMG. While our secondary outcomes included task performance, skin conductance, limb positioning, and difficulty/discomfort questionnaires.

A comprehensive and systematic electronic search was conducted from January 1, 1980, to March 1, 2024, on Cochrane Library, PUBMED, EMBASE, Google Scholar, and Web of Science, while adhering to the Preferred Reporting Items for Systematic Reviews and Meta-analyses (PRISMA) guidelines. We had no intention of searching the grey literature. Our search strategy consists of synonyms of box trainers and VR simulators. The search was restricted to English articles only. Similarly, due to the heterogeneity of methods in assessing ergonomics in laparoscopic training, we decided to select only papers utilizing EMGs and NASA-TLX, particularly the physical demand aspect of the questionnaire. Our objective was to identify ongoing and recently completed trials, encompassing randomized control trials (RCTs) and non-RCTs. Furthermore, extensive searching was conducted to find relevant articles in the references of included articles.

Study Selection

Articles obtained from literature searches and reference lists will be imported into ENDNOTE. Two reviewers independently evaluated each article’s eligibility and included studies on using box trainers or VR simulators for laparoscopic surgery training. Studies were excluded if they did not contain any ergonomic outcomes or were considered case series or studies. Any disparity between reviewers was addressed through discussion, until a consensus was reached.

Data Extraction

Data extraction and collection of primary and secondary outcomes were performed on an Excel spreadsheet (Microsoft® Corp., Redmond, WA, USA). Studies in which the results, particularly raw data, were not reported are still included in the review; however, the data from these studies may not be incorporated into the analysis, due to insufficient data.

Risk of Bias

In terms of risk of bias, two reviewers conducted independent evaluations of the risk of bias for each trial using the ROBINS-I (Risk Of Bias In Non-randomized Studies of Interventions) tool. Each component was assessed and graded according to its risk level: critical, serious, moderate, or low. If further information was required or missing, we contacted the relevant authors of the study. Any discrepancies or disagreements were resolved through consensus discussions between the two authors.

Results

**Figure 1:**
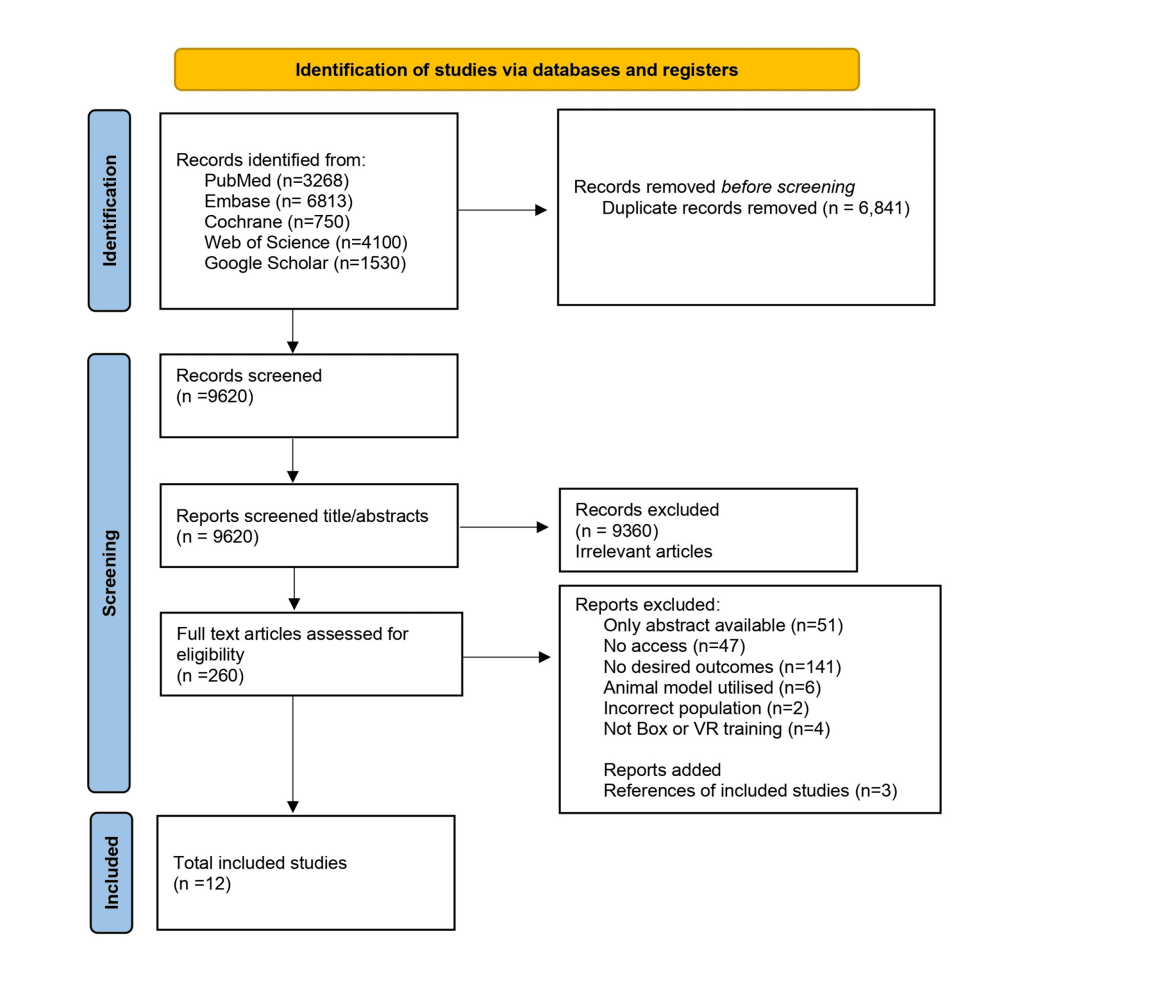
The PRISMA flowchart illustrates the process, and the study examines ergonomics in laparoscopic box training and VR simulation PRISMA: Preferred Reporting Items for Systematic Reviews and Meta-Analyses; VR: virtual reality

The screening process is depicted in the PRISMA (Preferred Reporting Items for Systematic Reviews and Meta-Analyses) flow diagram (Figure 1). The initial search identified a total of 16,461 articles. After the removal of 6,841 duplicate records and excluding 9,360 reports based on title and abstract, 260 articles remained for full-text evaluation. Subsequently, 251 articles were ruled out based on reasons delineated in Figure 1. Following a thorough full-text review, nine studies that satisfied the eligibility criteria were included in the systematic review. Furthermore, three additional studies were identified from the references of included studies, bringing the total number of included studies to 12. The incorporated studies encompassed a variety of study designs, as detailed in Table 1, and collectively involved 275 participants. The objective of this systematic review was to look at ergonomics in box trainers (141 participants) and VR laparoscopic training (134 participants). The primary outcomes included EMG in %MVC (maximum voluntary contraction) and µV (microvolts), as well as NASA-TLX scores. The results have been compiled into a table of primary and secondary outcomes shown in Tables 2-3, while the table of study characteristics can be seen in Table 1. Lastly, all findings are summarized in the summary of findings table, seen in Table 4.

**Table 1:** Study characteristics of ergonomics in laparoscopic box training and VR simulation EMG: electromyography; nRMS: normalized root mean square; VR: virtual reality; NASA-TLX: NASA Task Load Index; RULA: rapid upper limb assessment; SMEQ: subjective mental effort questionnaire; OSAT: objective structured assessment of technical skills

Author	Publication year	Study design	Participant (n)	Training level	Task	Comparator	Outcome measure (units)
Berquer et al. [7]	2002	Experimental, repeated measures	21	N/A	Cutting through a quarter-circle curved line on a piece of paper	Box trainer	EMG (µV), calculated upper arm elevation angle, skin conductance (micromhos), questionnaire to rate difficulty of task and musculoskeletal discomfort, performance time (s)
Uhrich et al. [8]	2002	Prospective observational	8	Attendings: 4; Resident trainees: 4	Point-to-point needle pass cylinder/Peg-Board needle drive/knot	Box trainer	EMG activity percentiles via nRMS amplitudes muscular discomfort scores were obtained before and after
Erden and Chun [9]	2018	Prospective observational	3	All novice	Hoop transfer game	Box trainer	Average number of transfers in a trial before and after training, number of disturbances, averaged muscle peak activity (µV) before and after training at pick-up and drop-off, and changes (%)
Nowakowski et al. [10]	2018	Prospective experimental	10	All medical students	Intracorporeal knot tying	Box trainer	Surface EMG (µV), number of knots tied in 15 minutes
Van veelan et al. [11]	2002	Prospective observational	8	N/A	Picking up chips with dissection forceps with an angled ring handle and placing these chips over the pins of an object	Box trainer	Discomfort questionnaire mean EMG% of neutral positions
Pérez-Duarte et al. [12]	2013	Prospective observational	30	Experts laparoscopic surgeon: 10; Novices: 20	Dissection suturing	Box trainer	Mean values of EMG activity (% of MVC)
Soto Rodriguez et al. [13]	2023	Prospective observational	14	Experts laparoscopic surgeon: 6; Medical students: 8	Circular pattern cutting task	Box trainer	Accuracy time taken to complete task EMG amplitude Muscle activity (iEMG) acceleration user movement fatigue
Bharathan et al. [14]	2013	Prospective observational	34	Experience: 9; Intermediate: 9; Novice: 16	Salpingectomy and salpingotomy	VR	NASA-TLX (includes data of physical demand); SMEQ; total time taken (s); total number of movements; total path length (cm)
Hu et al. [15]	2016	Prospective observational	47	Medical students	Ring transfer; Precision cutting; Intracorporeal suturing	Box trainer	Time taken to complete the task (s); accuracy; total test scores NASA-TLX
Youssef et al. [16]	2011	Experimental, repeated measures	8	Surgical residents: 5; MIS fellows: 2; Attending surgeon: 1	Cholecystectomy	VR	RULA; NASA-TLX; time taken; complication rate; number of clips applied; percentage of dissected fat use or misuse of electrocautery; subjective questionnaire
Nayar et al. [17]	2019	Prospective observational	41	Novice: 32; Experienced surgeons: 9	Appendectomy	VR	NASA-TLX SMEQ OSAT score (median); economy of left and right motion (average of path length/path time); electro-surgery or energy activation time (s); electro-surgery or energy usage while not in contact with tissue Idle time (s); injury to appendix; number of movements of left and right instrument; total path length of left and right instrument; time taken
Yu et al. [18]	2022	Prospective experimental	51	All medical students	Peg transfer; picking beans; threading skills; colon resection	VR	Performance scores pre and post-training; time taken to complete NASA-TLX WP scale; heart rate

**Table 2:** Primary outcomes for the ergonomics in laparoscopic box training and VR simulation VR: virtual reality; EMG: electromyography; NASA-TLX: NASA Task Load Index; nRMS: normalized root mean square

Author	Publication year	Muscles	EMG (p-values)	NASA-TLS (p-values)
Berquer et al. [7]	2002	Right deltoid, right trapezius	p < 0.05 for deltoid EMG at table heights of -10 cm and -20 cm; p < 0.05 for trapezius EMG at table heights of 10, 0, -10, -20	N/A
Uhrich et al. [8]	2002	Upper trapezius, sternocleidomastoid, middle trapezius, anterior deltoid, lower erector spinae, hamstrings	Mean nRMS for the upper trapezius was significantly higher with the view site compared to the standard monitor (p < 0.05); Mean nRMS for sternocleidomastoid was significantly higher with the standard monitor compared to the view site (p < 0.05); p < 0.05 for lower amplitudes seen in attending than resident surgeons for the sternocleidomastoid, middle trapezius, and hamstring	N/A
Erden and Chun [9]	2018	Lateral deltoid (LD), biceps, triceps, extensor digitorum (ED), flexor carpi radialis (FCR)	N/A	N/A
Nowakowski et al. [10]	2018	Thenar eminence, proximal forearm, deltoid muscle, trapezius muscle	p < 0.05 for decreases in the median values of surface EMG (sEMG) signals after the training period are visible for the non-dominant hand, deltoid, and trapezius muscle; p < 0.05 for an inverse correlation between higher skill and deltoid muscle activity	N/A
van Veelen et al. [11]	2002	Biceps brachii	N/A	N/A
Pérez-Duarte et al. [12]	2013	Trapezius, forearm flexors, forearm extensors	p < 0.01 for muscle activity was significantly lower in the expert group compared to the novice group in the trapezius muscle, forearm flexors (p < 0.005), and forearm extensors (p < 0.05); p < 0.01 for muscle activity during suturing was significantly higher compared to the dissection task in the trapezius muscle	N/A
Soto Rodriguez et al. [13]	2023	Trapezius, deltoid, biceps, pectoralis major, forearm flexors	Trapezius: left p-value 0.002, right p-value 0.08; Deltoid: Left p-value < 0.001, right p-value < 0.001; Biceps: left p-value 0.78, right p-value 0.70; Pectoralis major: left p-value 0.78, right p-value 0.70; Forearm flexors: left p-value 0.003, right p-value 0.007	N/A
Bharathan et al. [14]	2013	N/A	N/A	N/A
Hu et al. [15]	2016	N/A	N/A	p < 0.001 for decrease in NASA-TLX scores after training for all tasks; p < 0.001 for increase in NASA-TLX with increased complexity of the task
Youssef et al. [16]	2011	N/A	N/A	Side and between standing: physical (p = 0.001); Effort (p < 0.05); Performance p-value: not significant
Nayar et al. [17]	2019	N/A	N/A	Physical demand (p-value: 0.27); Performance (p-value: 0.82); Effort (p-value: 0.17)
Yu et al. [18]	2022	N/A	N/A	Cognitive load scores were significantly reduced for both fundamental surgery tasks and colon resection tasks (p < 0.05 for fundamental surgery, p < 0.01 for colon resection)

**Table 3:** Secondary outcomes for the ergonomics in laparoscopic box training and VR simulation VR: virtual reality; VRLS: virtual reality laparoscopic simulator

Author	Publication year	Muscles	Skin conductance	Task performance	Limb positioning	Questionnaire
Berquer et al. [7]	2002	Right deltoid, right trapezius	p > 0.05	p > 0.05, in terms of performance time	p < 0.05 for the reduction in upper arm elevation at lower table heights	p = 0.054 in terms of rating of discomfort, p < 0.05 in terms of discomfort at 0- and +10 cm table height compared to very low or high table heights
Uhrich et al. [8]	2002	Upper trapezius, sternocleidomastoid, middle trapezius, anterior deltoid, lower erector spinae, hamstrings	N/A	N/A	N/A	p < 0.05 for subjective discomfort questionnaire scores between attending and resident surgeons, with residents experiencing greater discomfort in certain muscles, including the lower erector spinae and left hamstring
Erden and Chun [9]	2018	Lateral deltoid (LD), biceps, triceps, extensor digitorum (ED), flexor carpi radialis (FCR)	N/A	N/A	N/A	N/A
Nowakowski et al. [10]	2018	Thenar eminence, proximal forearm, deltoid muscle, trapezius muscle	N/A	N/A	N/A	N/A
van Veelen et al. [11]	2002	Biceps brachii	N/A	N/A	Height of the operating table at 0.5, 0.6, 0.7, or 0.8 of the elbow height; more neutral shoulder positions were recorded than with the factors 0.9 and 1.0 (p < 0.05); Factors 0.7 and 0.8 of the elbow height scored significantly better than factors 0.5, 0.6, 0.9, and 1.0: more neutral elbow excursions were recorded (p < 0.05)	Discomfort score: mean of 64.3 (SD 20)
Pérez-Duarte et al. [12]	2013	Trapezius, forearm flexors, forearm extensors	N/A	N/A	N/A	N/A
Soto Rodriguez et al. [13]	2023	trapezius, deltoid, biceps pectoralis major, forearm flexors	N/A	p < 0.001 for the circular pattern cutting task in cutting area accuracy between experts and novices; p < 0.001 for time taken to complete the task between experts and novices	N/A	N/A
Bharathan et al. [14]	2013	N/A	N/A	Time taken: p < 0.001 for experienced vs. intermediate vs. novice; Total number of movements: p = 0.005 for experienced vs. intermediate vs. novice; Path length: p = 0.055 for experienced vs. intermediate vs. novice	N/A	N/A
Hu et al. [15]	2016	N/A	N/A	p < 0.001 for total test scores, which decreased significantly after training	N/A	N/A
Youssef et al. [16]	2011	N/A	N/A	N/A	N/A	N/A
Nayar et al. [17]	2019	N/A	N/A	Time taken to complete the procedure (p = 0.0039); total number of movements in each hand (right hand: p < 0.0001; left hand: p < 0.0001); number of injuries to the appendix (p = 0.0022); idle time (p = 0.0006)	N/A	N/A
Yu et al. [18]	2022	N/A	N/A	p < 0.01 for time taken by participants to complete tasks on VRLS, which significantly decreased compared to pre-test values; p < 0.01 for improvement in performance scores for both fundamental surgery tasks and colon resection tasks	N/A	N/A

**Table 4:** Summary of findings table for the ergonomics in laparoscopic box training and VR simulation The risk in the intervention group (and its 95% CI) is based on the assumed risk in the comparison group and the relative effect of the intervention (and its 95% CI). GRADE Working Group grades of evidence - High Certainty: We are very confident that the true effect lies close to that of the estimate of the effect. Moderate Certainty: We are moderately confident in the effect estimate; the true effect is likely to be close to the estimate of the effect, but there is a possibility that it is substantially different. Low Certainty: Our confidence in the effect estimate is limited; the true effect may be substantially different from the estimate of the effect. Very Low Certainty: We have very little confidence in the effect estimate; the true effect is likely to be substantially different from the estimate of the effect. ^a^Studies ranged from moderate to serious risk of bias; ^b^Outcome was measured differently across the studies; ^c^No confidence interval was conducted therefore the effect of precision is not known; ^d^The study reporting this outcome has a serious risk of bias; ^e^Both studies reported a serious risk of bias; ^f^Studies ranged from critical to moderate risk of bias; ^g^High risk of selection bias as well as poor explanations of interventions utilized in the studies; ^h^Most studies observed a significant p-value in terms of NASA-TLX, however, one of the studies with a moderate risk of bias found no significance; ^i^Studies were conducted in a setting that reflected current practice with simulators that are still utilized among trainees; ^j^Outcomes were measured differently in terms of units of EMG and although the majority of the results were in favor of improved ergonomics leading to improved task performance, one study contradicted this finding. CI: confidence interval; EMG: electromyography; NASA-TLX: NASA Task Load Index; VR: virtual reality

Outcomes	No of participants (studies) follow-up	Certainty of the evidence (GRADE)	Impact
				Objective ergonomics measurement assessed with: EMG	94 (6 non-randomized studies)	⨁⨁◯◯ denotes Low^a,c,j^	Overall, most studies found an increase in task performance as ergonomics improved. Training level was indirectly correlated to the level of muscle activity as well.
Subjective questionnaire assessed with: NASA-TLX	181 (5 non-randomized studies)	⨁◯◯◯ denotes Very low^g,h,i^	3/4 studies found a significant decrease in NASA-TLX scores with box training and VR simulators. While one of the studies had non-significant findings in terms of NASA-TLX post-training on a VR simulator.
Performance task	208 (6 non-randomized studies)	⨁⨁◯◯ denotes Low^a,b,c^	All box trainer studies except one had significant findings in terms of task performance with a p-value of >0.05. On the other hand, VR simulators, all studies had significant improvement in task performance with a p-value < 0.01.
Skin conductance	21 (1 non-randomized study)	⨁⨁⨁◯ denotes Moderate^c,d^	One study on box trainers found a significant increase in skin conductance with poor ergonomics. None of the VR studies reported skin conductance.
Limb positioning	29 (2 non-randomized studies)	⨁⨁◯◯ denotes Low^b,c,e^	In both studies, one involving box trainers and the other utilizing VR simulation, a p-value < 0.05 was observed for a reduction in non-neutral limb position in poor ergonomic settings.
Subjective (non-NASA-TLX) questionnaire	36 (3 non-randomized studies)	⨁◯◯◯ denotes Very low^b,c,f^	All studies were on box trainers with the majority of them reporting a p-value of <0.05 for discomfort in poor ergonomic settings. A singular study also reported a significant increase in discomfort with lower training levels.

Risk of Bias

**Figure 2:**
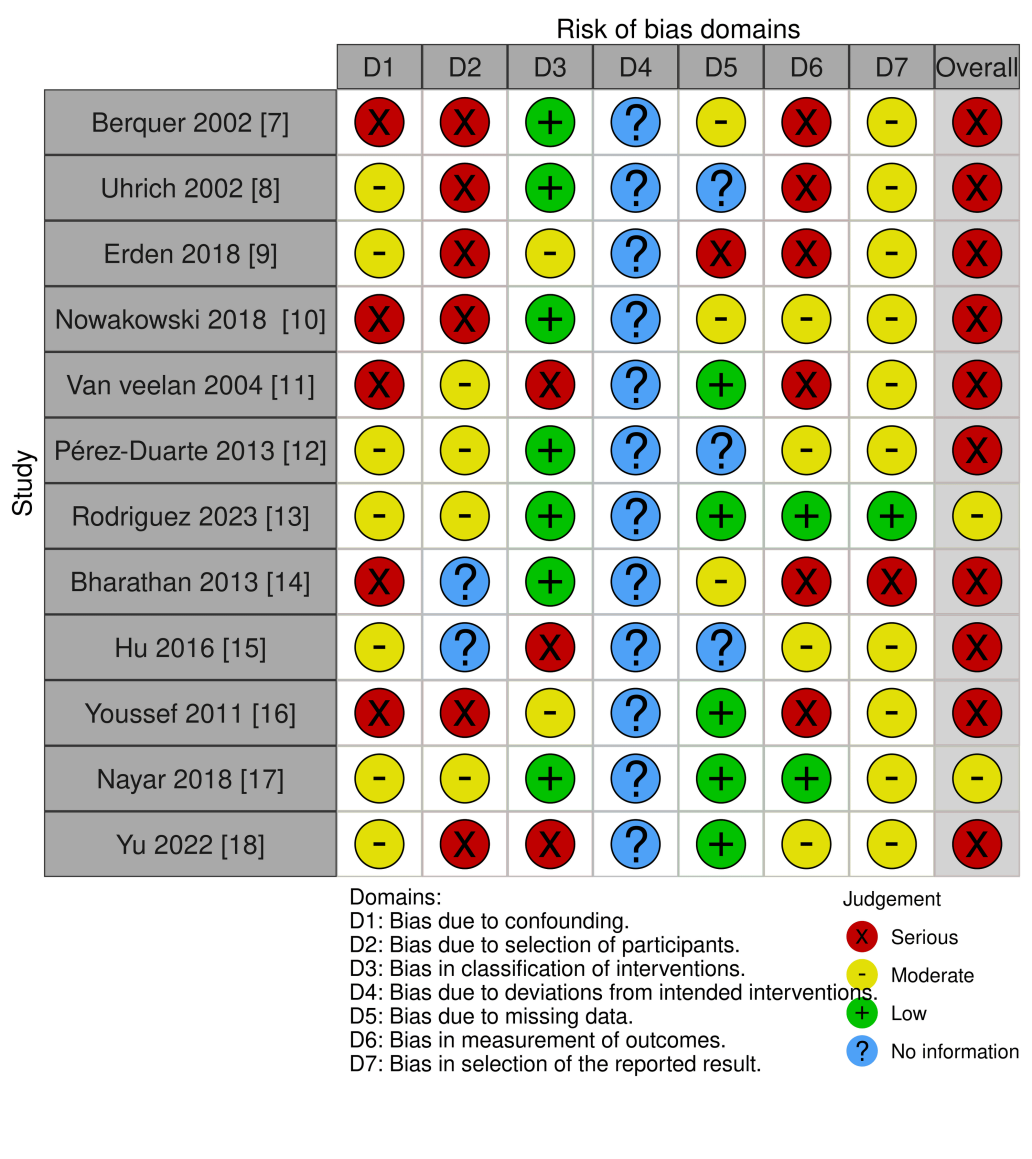
The ROBINS-I tool was used to assess the ergonomics in laparoscopic box training and VR simulation ROBINS-I: Risk Of Bias In Non-randomized Studies - of Interventions; VR: virtual reality

The studies included in the analysis were assessed for risk of bias, ranging from moderate to serious. The majority of studies were categorized under serious risk of bias, indicating significant concerns with their design or execution. These issues may affect the reliability of the findings. The full risk of bias assessment is illustrated in Figure 2, which provides a visual summary of the risk levels across the studies.

Participants

Participants varied in training levels from medical students to surgeons from different specialties, including gynecologists, urologists, and general surgeons.

Primary Outcome

EMG results: As part of the analysis, 6 of the 12 studies reported EMG results [7-10,12,13]. Due to variations in the methods of reporting EMG outcomes, the results were categorized into %MVC, normalized root mean square (nRMS), and cumulative workload (CMW). Five studies presented their EMG findings in terms of MVC and µV, exclusively utilizing box trainers. Regarding NASA-TLX, generally across the other studies, there appeared to be a negative correlation between participants' skills and muscle activity in the deltoids, forearms, extensor digitorum (ED), and flexor carpi radialis (FCR). On the other hand, there was a slight discrepancy regarding the forearm muscles; Erden and Chun [9] found an increase in FCR activity with greater skill, possibly due to the necessity for increased stiffness to enhance precision during task performance.

NASA-TLX: Out of the manuscripts reviewed, five reported NASA-TLX results, with one focusing on box trainers and four on VR systems [14-18]. The studies examining box trainers noted a significant difference in the correlation between task complexity and NASA-TLX scores, as well as a significant reduction in physical demand after training. Similarly, participants reported higher physical demand with box trainers, consistent with the EMG results. VR trainers also yielded similar findings, where increased dexterity and training were associated with a decrease in cognitive load. Overall, both box training and VR training produced similar outcomes, although the extrapolated results may be inconclusive due to the heterogeneity among the studies.

Secondary Outcome

Task performance: Out of the six studies reporting task performance, several noted a negative correlation between performance, training, and training levels in both box and VR trainers [7,13-15,17,18]. Significant differences were observed between experts and novices in a VR appendicectomy scenario, with fewer median numbers of injuries to the appendix, shorter idle time, and decreased time taken to complete the task.

Skin conductance: Berquer et al. reported skin conductance as an alternative way to measure ergonomics [7]. It was observed that a significant p-value was found, indicating that poor ergonomics at different table heights in box trainers can lead to increased effort and skin conductance. However, out of the 12 studies selected, only one reported this outcome; therefore, further research is needed to confirm this.

Limb positioning: In terms of limb position, two studies reported this outcome [7,11]. It was found that, in these studies, poor ergonomics at table levels that were either too high or too low were linked with less neutral positioning and improper angling of the arms.

Questionnaire: Overall, 5 of the 12 studies reported participants' feelings of discomfort and task difficulty via a questionnaire [7,8,11,13,16]. Generally, it was found that feelings of discomfort were inversely correlated to training levels; this finding was significant, with a higher score of discomfort found in surgeons who were residents compared to attendings.

Discussion 

While laparoscopic surgery offers advantages for patients, it poses risks for surgeons, as difficulties in maintaining proper ergonomics can lead to musculoskeletal injuries. Alarmingly, a study revealed that 87% of laparoscopic surgeons reported experiencing musculoskeletal pain [12]. Laparoscopic surgeons often adopt positions involving exaggerated ulnar deviation, wrist flexion, and arm abduction while using surgical tools, which can result in paresthesia and finger numbness due to compression of the digital nerve, known as "laparoscopic surgeon's thumb" [12].

Despite the prevalence of these issues, surgical ergonomics education remains uncommon. A survey conducted among program directors across 14 surgical specialties accredited by the Accreditation Council for Graduate Medical Education (ACGME) revealed that only 1.5% of respondents were provided with formal ergonomic training, while only 25.4% were provided with informal ergonomic training [13]. To our knowledge, this is the first systematic review conducted to assess ergonomics in both box and VR training. The aggregated data from included studies suggest a consistent trend: a reduction in muscle activity and physical demand among experts or those who have undergone training, regardless of whether it was VR or box training. Furthermore, there appears to be a correlation between performance and ergonomics, with participants achieving higher performance scores reporting lower levels of physical demand.

Likewise, Xiao et al.'s research found similar findings, demonstrating that an optimal ergonomic setting, characterized by greater adherence to neutral positions and reduced joint excursion, correlates with better task performance [5]. Therefore, it is recommended to uphold a neutral posture within the skills lab during training sessions. The American College of Surgeons has emphasized that an increased risk of musculoskeletal injuries can significantly affect surgeons' performance quality. This impact may stem from negative effects on cognitive processes, heightened fatigue, and accumulated stress experienced by surgeons [19].

In our systematic review, we observed that laparoscopic training tends to be associated with higher fatigue levels, a well-documented phenomenon in both existing literature and operating theaters, attributable to the awkward position involving greater elbow and wrist flexion [10]. In a study conducted by van der Schatte Olivier et al., it was observed that this modality resulted in reduced cognitive workload and physical discomfort, ultimately leading to significantly improved performance across all tasks, with fewer failures [20].

There are a few limitations to our study. Firstly, there was an absence of higher-quality studies on ergonomics in VR and box training, and most studies included in the systematic review contained a high risk of bias. Therefore, the study lacks high-quality evidence, with a huge potential for confounding variables that may influence the observed outcomes, decreasing confidence in establishing causality between ergonomics and laparoscopic training. Due to the heterogeneity of tasks, the method of measuring outcomes, and the data of the studies included, we were unable to carry out a meta-analysis. This means that we were unable to provide a comprehensive overview of quantitative evidence with more precise estimates of effect sizes and measures of variability. The absence of a search in the grey literature means that unpublished trials may have been overlooked, potentially introducing publication bias into this study. This bias could skew the results when assessing ergonomics in box or VR training.

Additionally, the studies conducted on ergonomics in box and VR training are limited, restricting the sample size and potentially affecting the external validity of results. Despite showcasing mostly consistent results, the data from this systematic review should be interpreted with caution. For further evidence, it is advisable to standardize the measurement of ergonomics and strive for higher-quality evidence, particularly through the inclusion of randomized controlled trials (RCTs), in order to validate ergonomic outcomes associated with box and VR training for laparoscopic surgeons. This would enhance the reliability and validity of findings, leading to more informed decision-making in surgical training and practice.

## Conclusions

In conclusion, there is reduced muscle activity and physical demand among trained individuals, with a correlation between higher performance and lower physical strain in both box and VR laparoscopic training. However, it's important to acknowledge that there is a lack of high-quality studies looking at VR and box training, leaving the evidence on this matter inconclusive.
